# Preimplantation factor is an anti-apoptotic effector in human trophoblasts involving p53 signaling pathway

**DOI:** 10.1038/cddis.2016.382

**Published:** 2016-12-01

**Authors:** Hadia Moindjie, Esther Dos Santos, Rita-Josiane Gouesse, Nelly Swierkowski-Blanchard, Valérie Serazin, Eytan R Barnea, François Vialard, Marie-Noëlle Dieudonné

**Affiliations:** 1 GIG-EA7404, Université de Versailles Saint-Quentin-en-Yvelines – Paris Saclay, Unité de Formation et de Recherche des Sciences de la Santé-Simone Veil, Montigny-le Bretonneux, France; 2 Service de Biologie Médicale, Centre Hospitalier de Poissy-Saint Germain, Poissy, France; 3 Département de Biologie de la Reproduction, Cytogénétique, Gynécologie et Obstétrique, Centre Hospitalier de Poissy-Saint Germain, Poissy, France; 4 Society for the Investigation of Early Pregnancy, Cherry Hill, NJ, USA; 5 BioIncept, LLC, Cherry Hill, NJ, USA

## Abstract

From the earliest stages of gestation, embryonic–maternal interaction has a key role in a successful pregnancy. Various factors present during gestation may significantly influence this type of juxta/paracrine interaction. PreImplantation Factor (PIF) is a recently identified factor with activity at the fetomaternal interface. PIF is secreted by viable embryos and directly controls placental development by increasing the invasive capacity of human extravillous trophoblasts (EVTs). To further specify PIF's role in the human placenta, we analyzed the genome-wide expression profile of the EVT in the presence of a synthetic PIF analog (sPIF). We found that sPIF exposure altered several pathways related to p53 signaling, survival and the immune response. Functional assays revealed that sPIF acts through the p53 pathway to reduce both early and late trophoblast apoptosis. More precisely, sPIF (i) decreases the phosphorylation of p53 at Ser-15, (ii) enhances the B-cell lymphoma-2 (BCL2) expression and (iii) reduces the BCL2-associated X protein (BAX) and BCL2 homologous antagonist killer (BAK) mRNA expression levels. Furthermore, invalidation experiments of TP53 allowed us to demonstrate that PIF's effects on placental apoptosis seemed to be essentially mediated by this gene. We have clearly shown that p53 and sPIF pathways could interact in human trophoblast and thus promotes cell survival. Furthermore, sPIF was found to regulate a gene network related to immune tolerance in the EVT, which emphasizes the beneficial effect of this peptide on the human placenta. Finally, the PIF protein levels in placentas from pregnancies affected by preeclampsia or intra-uterine growth restriction were significantly lower than in gestational age-matched control placentas. Taken as a whole, our results suggest that sPIF protects the EVT's functional status through a variety of mechanisms. Clinical application of sPIF in the treatment of disorders of early pregnancy can be envisioned.

Placentation is a critical step in the establishment of a successful pregnancy. The placenta is a transitory organ responsible for fetomaternal exchanges and maternal immunotolerance. The chorionic villi constitute the structural and functional units of the placenta. The mature chorionic villus is delimited by a double layer of epithelial cells, the mononuclear villous cytotrophoblast (CTV) and the multinucleate syncytiotrophoblast (ST).^1, 2^ The extravillous trophoblast (EVT) has invasive properties that are essential for implantation and uterine artery remodeling.^3, 4^ Trophoblast invasion is a highly restricted and regulated process with a pivotal role in the development and progression of pregnancy. However, the molecular mechanisms involved in EVT invasion have not been extensively characterized.

Herein, we focused on preimplantation factor (PIF), a 15-amino-acid peptide (MVRIKPGSANKPSDD) secreted by viable, developing embryos.^5, 6^ It is now well established that PIF exerts autotrophic and protective effects on the embryo.^7, 8, 9^ Furthermore, PIF is also detected in the maternal circulation throughout pregnancy. The presence of PIF in maternal serum has been correlated with live births in murine and bovine models.^10^ PIF also regulates the maternal environment by promoting human endometrial receptivity. In human decidual cells, PIF exerts pro-apoptotic effects and creates a beneficial pro-inflammatory environment.^11, 12^ Moreover, PIF orchestrates maternal systemic immune responses.^13^ Pathway analysis in models of autoimmunity and transplantation have demonstrated that when administered as a single agent to non-pregnant mice, synthetic PIF analog (sPIF) is associated with a reduction in oxidative stress^8, 14^ and protein misfolding.^15, 16, 17, 18, 19^ Finally, PIF is expressed by the placenta and by hematopoietic fetal tissues.^13, 20^ Moindjie *et al.* have recently reported that PIF expression in trophoblastic cells is prominent in the earliest stages of pregnancy and then declines at term. This observation suggests that endogenous PIF has a significant role in the critical postimplantation phase during which development of the trophoblast must be regulated. Effectively, sPIF was shown to promote invasion in human HTR-8/SVneo trophoblastic cell line.^21^ Recently, we reported that sPIF also increases human EVT invasion without affecting cell proliferation.^14, 20^

Coordinated proliferation, differentiation and death of trophoblastic cells are required for the development of a functional placenta. Programmed cell death is an active process required for normal trophoblastic cell turnover.^22, 23, 24^ The tumor-suppressor gene *TP53* is a key component in cell cycle progression and the induction of apoptosis. p53 protein is an important transcription factor that regulates growth arrest, apoptosis and DNA repair in response to various stress stimuli.^25^ Upon these cellular stresses, p53 is phosphorylated and acetylated at multiple sites to activate downstream target genes. p53 induces its own negative feedback loop by stimulating the expression of mouse double minute 2 homolog (MDM2), which directly promotes p53 degradation by proteasome.^26^ It is well established that phosphorylation of p53 at Ser-15 leads to the dissociation between MDM2 and p53 and then results to p53 stabilization.^25^ Consequently, Ser-15 phosphorylation contributes to the preferential transactivation of pro-apoptotic genes.^27^ Among its target genes, p53 regulates the expression of the B-cell lymphoma-2 (BCL2) family proteins, which have a crucial role in apoptosis induced through a mitochondria mediated intrinsic pathway. Indeed, p53 stimulates the expression of various cell death inducers, such as BCL2-associated X protein (BAX), and BAK and represses expression of BCL2, a pivotal cell death inhibitor. As BAX and BAK are essential pro-apoptotic mitochondrial proteins responsible for the permeabilization of the mitochondrial outer membrane, an upregulation of BAX and BAK results in the activation of caspase cascade. These modifications result to morphological and biochemical changes associated with apoptosis.^28^ Interestingly, a weak invasiveness and elevated levels of villous trophoblast apoptosis have been described in preeclampsia (PE)^29, 30^ and in intra-uterine growth restriction (IUGR).^31, 32^

Although detailed studies of the decidua and systemic immunity have already revealed the mechanisms involved in PIF's targeted actions, the molecular mechanisms underlying PIF's effects in human trophoblasts have not been well identified.

In this study, we examined whole-genome gene expression profiles of human primary EVT cultured in the presence or absence of sPIF. Bioinformatic analysis suggested that sPIF has a role in the control of apoptosis and immunity in human trophoblast. By using functional assays, we further demonstrated that sPIF exerts a direct anti-apoptotic effect on human EVT. Finally, we demonstrated that PIF protein levels in the human placental villi from IUGR and PE pregnancies are significantly lower than in gestational age-matched control placentas. These results suggest that the dysregulation of PIF protein expression may be associated with disorders of pregnancy.

## Results

### Gene expression profiles in sPIF-treated human EVT

**Figure 1:**
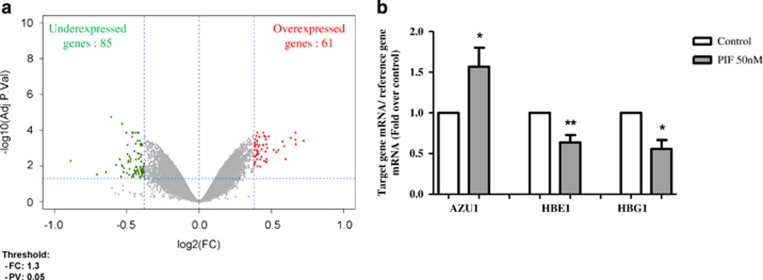
Transcriptomic analysis of the genes differentially regulated by sPIF in EVT. (**a**) A volcano plot of the FC *versus* the adjusted *P*-value for the DEGs in EVT cultured in the absence (control) or presence of sPIF 50 nM for 24 h. The two vertical lines correspond to FC=1.3 and the horizontal line corresponds to *P*<0.05. Overexpressed transcripts are shown in red and underexpressed transcripts are shown in green. (**b**) Quantitative real-time PCR analysis of selected transcripts differentially regulated by sPIF in EVT. EVT were cultured in the absence (control) or presence of sPIF (50 nM) for 24 h. Total RNA was extracted and analyzed by RT-PCR, as described in Materials and Methods section. Values are the mean±S.E.M. of six to eight independent experiments and are expressed as fold over control. **P<0.01; *P<0.05. Wilcoxon test. *HBE1*, hemoglobin epsilon 1; *HBG1*, hemoglobin gamma 1

To study the mechanisms possibly affected by treatment with sPIF, we performed a genome-wide microarray analysis of human EVT cultured in the absence (control) or presence of sPIF (50 nM) using an Agilent 8 × 60k microarray. This analysis identified groups of genes that were significantly over- or underexpressed (relative to controls) after sPIF treatment of EVT. We opted for an adjusted *P-*value threshold <0.05 and a fold-change (FC) cut-off of 1.3. As shown in Figure 1a, sPIF treatment of EVT was associated with significant changes in the expression level of 146 genes (85 downregulated and 61 upregulated). In particular, sPIF upregulated the expression of (i) the gene coding for azurocidin-1 (AZU1), a protein with an important role in the chemotactic and antimicrobial activity of monocytes, (ii) the gene coding for olfactory receptor family 10 subfamily A member 7 (OR10A7), (iii) interleukin 17F (IL17F) and (iv) two long intergenic noncoding RNAs (lincRNAs XLOC-006051 and XLOC-004148). Treatment with sPIF also downregulated the fetal gamma and epsilon globin genes (HBG1 and HBE1, respectively, which are expressed in the yolk sac and turned down after trophoblast differentiation). Gene names, (FC) and adjusted *P*-value for each over- or underexpressed gene are described in Table 1.

To confirm the gene chip data, we used quantitative real-time PCRs to analyze (i) transcripts of the most upregulated gene (AZU1) and (ii) transcripts of the two most downregulated genes (HBG1 and HBE1). The expression patterns are correlated with the microarray profiling data (Figure 1b).

### Functional networks regulated by sPIF in treated human EVT

**Figure 2:**
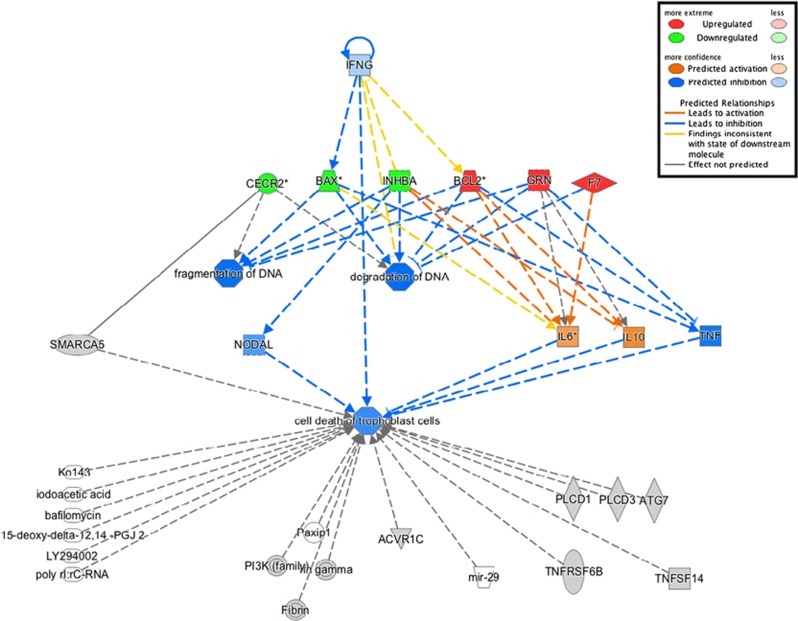
The gene network associated with ‘cell death and survival, DNA replication, recombination, and repair' in sPIF-treated EVT. Green shapes present downregulated genes and red shapes present upregulated genes. Predicted relationships are indicated on the figure

First, genes with altered expression in sPIF-treated EVT were analyzed using QIAGEN's Ingenuity Pathway Analysis (IPA) software (Ingenuity Systems, Redwood City, CA, USA). Among the top molecular and cellular functions, ‘cancer', ‘cellular development, growth and proliferation' and ‘cell death and survival, DNA replication, recombination, and repair' functions were identified, with *P*-values of 3.07 × 10^−2^, 3.33 × 10^−2^ and 5.84 × 10^−4^, respectively. Changes in expression after sPIF treatment were observed for 34 genes in the ‘cancer' subcategory, 14 genes in the ‘cellular development, growth, and proliferation' subcategory and 6 genes in the ‘cell death and survival, DNA replication, recombination and repair' subcategory. In the latter gene network shown in Figure 2, sPIF specifically downregulated *BAX*, *CECR2* (coding for Cat Eye Syndrome Chromosome Region, Candidate 2), *INHBA* (coding for inhibin beta A) and upregulated *BCL2*, *F7* (coding for coagulation factor VII) and *GRN* (coding for granulin) in human EVT. All these genes are related to DNA degradation, DNA fragmentation and tumorigenic processes, and three of them are under the control of interferon gamma (IFNG). However, the FC for the *IFNG* gene (−1.05) was not statistically significant in this experiment.

**Figure 3:**
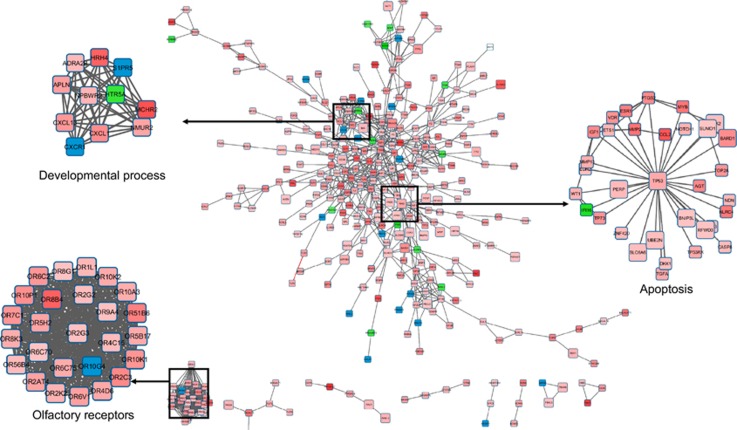
A PPI network analysis. The DEGs identified in the microarray analysis were used to build a PPI network. The STRING database was used to screen interacting proteins, which were then visualized as a PPI network using Cytoscape software. The network nodes represent genes and the edges represented interactions. The size of the nodes is proportional to the number of connections established with other genes. The ‘developmental process', ‘olfactory receptors' and ‘apoptosis' clusters are presented on a larger scale. The gene expression levels were mapped on the network. Red indicates upregulation and green downregulation

In order to extract further biological informations, we performed a gene set enrichment analysis (GSEA) of the whole-gene expression data set and found 478 significantly enriched gene sets (false discovery rate (FDR) <0.25). Critical keywords for the gene sets were ‘cancer', ‘immunity' and ‘ion channel transport'. The ‘cancer' gene set was downregulated, and the ‘immunity' and ‘ion channel transport' gene sets were upregulated (data not shown). Differentially expressed genes (DEGs) were analyzed with the Database for Annotation, Visualization, and Integrated Discovery (DAVID). The protein–protein interaction (PPI) relationship was obtained from STRING database, and the PPI network and the functional modules were visualized using Cytoscape (Figure 3). The PPI network contained 424 nodes (representing the DEGs) and 1045 edges (representing the interactions between the DEGs). Next, we used the Kyoto Encyclopedia of Genes and Genomes (KEGG) database to link higher-order biological information to the genomic information associated with these distinct genes and sPIF treatment. The distinct functional categories for the sPIF-associated gene lists are shown in Table 2. Our analysis showed that a few enriched KEGG pathways were related to the ‘immune system' and ‘viral infection', such as *tuberculosis, graft-versus-host disease, type I diabetes mellitus* and *autoimmune thyroid disease.* In confirmation of the gene expression profile data shown in Table 1, sPIF treatment in EVT seems to affect *olfactory signal transduction.* Finally, the observed expression changes seem to be highly associated with ‘tumorigenesis pathways', such as *pathways in cancer, apoptosis,* and *p53 signaling pathway*.

### Effect of sPIF on human trophoblast viability and apoptosis

**Figure 4:**
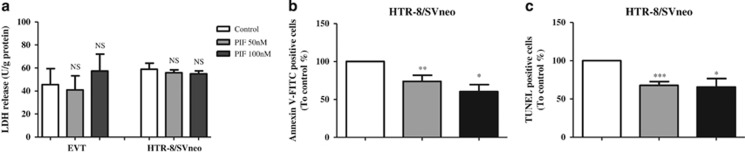
Effect of sPIF on cell viability and apoptosis. (**a**) LDH levels in the culture medium for human primary EVT and HTR-8/SVneo cells after a 24-h incubation in the absence (control) or presence of sPIF (50 and 100 nM). The values are the mean±S.E.M. of four independent experiments. (**b**) Annexin V-FITC assay of HTR-8/SVneo cells after 48 h of culture in the absence (control) or presence of sPIF (50 or 100 nM). The values are the mean±S.E.M. from 10 independent experiments and are expressed as a percentage of the control. (**c**) TUNEL staining in HTR-8/SVneo cells after 48 h of culture in the absence (control) or presence of sPIF (50 or 100 nM). The values are the mean±S.E.M. from 6 to 11 independent experiments and are expressed as a percentage of the control. ***P<0.001; **P<0.01; *P<0.05, and NS, nonsignificant. Wilcoxon test

To confirm sPIF's effects on apoptosis pathway in human trophoblasts, we performed a number of functional assays. We first used lactate dehydrogenase (LDH) activity and Trypan blue exclusion assays to ascertain viability in EVT and HTR-8/SVneo cells. Our results showed that sPIF (at 50 or 100 nM) did not affect LDH activity in either cell type (Figure 4a). The Trypan blue exclusion assay confirmed that sPIF did not significantly affect the viability of HTR-8/SVneo cell (87%±1.5, 90%±1.2, and 92%±0.7 for the control, 50 nM sPIF and 100 nM sPIF conditions, respectively; *n*=4 for each). Next, we studied the effect of sPIF on the early steps of apoptosis with a flow cytometric annexin V-FITC staining assay. As shown in Figure 4b, exposure of HTR-8/SVneo cells to sPIF (at 50 and 100 nM) was associated with lower levels of cell apoptosis (by 26.3% and 39.6%, respectively). Second, we examined the effects of sPIF on trophoblast DNA fragmentation (the last step of programmed cell death) in a terminal deoxynucleotidyl transferase-mediated dUTP-biotin DNA-nick end labeling (TUNEL) assay. As shown in Figure 4c, sPIF (at 50 and 100 nM) was associated with a significant lower apoptotic index (by 32.2% and 34.4%, respectively) in HTR-8/SVneo cells.

### Effects of sPIF on TP53 mRNA expression, p53 protein levels and p53 activation in human trophoblasts

**Figure 5:**
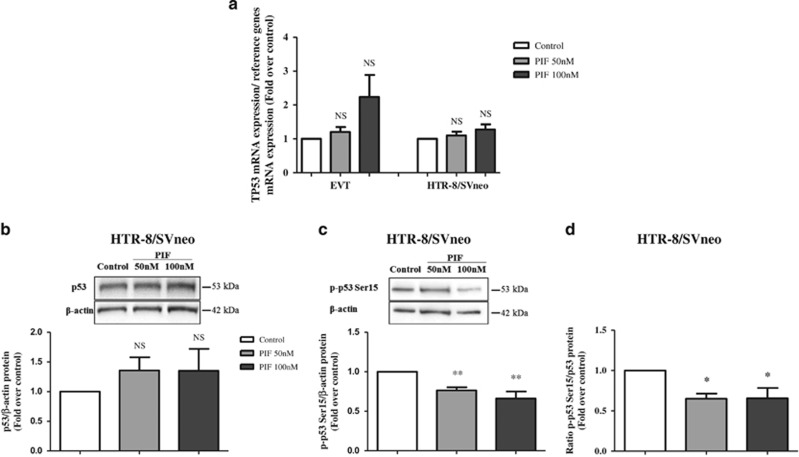
Effect of sPIF on TP53 mRNA expression, p53 protein levels and p53 activation in human trophoblasts. (**a**) TP53 mRNA expression in trophoblastic cells cultured for 24 h in the absence (control) or presence of sPIF (50 or 100 nM). Total RNA was extracted and analyzed by RT-PCR, as described in Materials and methods section. The values are the mean±S.E.M. from six to nine independent experiments and are expressed as fold over control. (**b**) p53 protein expression and (**c**) phospho-p53 (Ser-15) protein expression were quantified by western blotting, as described in Materials and methods section. (**d**) The p-p53 Ser-15/p53 protein ratio. Total protein was extracted from HTR-8/SVneo cells cultured for 24 h in the absence (control) or presence of sPIF (50 or 100 nM). The values are the mean±S.E.M. from nine independent experiments and are expressed as fold over control. **P<0.01; *P<0.05; NS, nonsignificant. Wilcoxon test

In order to specify the molecular mechanisms involved in sPIF's anti-apoptotic effect, we examined the status of p53 (expression and phosphorylation) in trophoblast cells. First, we measured p53 expression in EVT and HTR-8/SVneo cells. In both cell types, sPIF did not affect TP53 mRNA levels (Figure 5a). p53 protein levels were not modified by treatment with sPIF in HTR-8/SVneo cells (Figure 5b). The post-translational modification at Ser-15 was shown to be associated with increased p53 stability and transcriptional activity. Then, we have decided to evaluate the effect of sPIF on the Ser-15 phosphorylation state of p53. In the presence of sPIF at 50 and 100 nM, the Ser-15 phospho-p53 expression was significantly reduced in HTR-8/SVneo cells (by 24% and 34%, respectively; Figure 5c). Finally in HTR-8/SVneo cells, we have showed that sPIF treatment decreased the phospho-p53 Ser-15/p53 protein ratio (by 35% at 50 and 100 nM; Figure 5d).

### Effect of sPIF on p53 transcriptional activity in human trophoblasts

**Figure 6:**
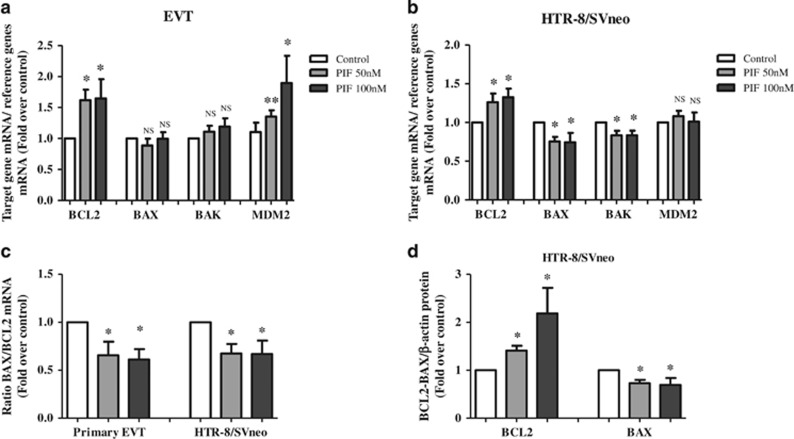
Effect of sPIF on transcriptional targets of p53. BCL2, BAX, BAK and MDM2 mRNA expression levels in (**a**) primary EVT and (**b**) HTR-8/SVneo cells after 24 h of culture in the absence (control) or presence of sPIF (50 or 100 nM). Total RNA was extracted and analyzed by RT-PCR, as described in Materials and methods section. (**c**) The BAX/BCL2 mRNA ratio. The values are the mean±S.E.M. from six to eight independent experiments and are expressed as fold over control. (**d**) BCL2 and BAX protein expression levels in HTR-8/SVneo cells were quantified by western blotting, as described in Materials and methods section. The values are the mean±S.E.M. from five independent experiments and are expressed as fold over control *P<0.05; **P<0.01; NS, nonsignificant. Wilcoxon test

Given that bioinformatics analyses have suggested that sPIF regulated the p53 signaling pathway, we studied the mRNA expression levels of four direct targets of p53 (BCL2, BAX, BAK and MDM2). In EVT, exposure to 50 and 100 nM sPIF resulted in a significant increase of MDM2 (by 35% and 89%, respectively), and BCL2 gene expression (by 62% and 64%, respectively). In contrast, sPIF did not affect BAX and BAK gene expression levels under the same experimental conditions, suggesting that these genes are not directly implicated in sPIF anti-apoptotic action in EVT (Figure 6a). As shown in Figure 6b, treatment of HTR-8/SVneo cells with 50 and 100 nM sPIF was associated with elevated levels of BCL2 mRNA (with relative increases of 26% and 32%, respectively) and lower of BAX (by 25% at both doses), and BAK (by 17% at 50 and 100 nM) mRNA levels. sPIF treatment was associated with significant decrease of BAX/BCL2 mRNA ratio in both EVT (by 39% at both doses) and HTR-8/SVneo cells (by 34% at both doses; Figure 6c). Finally, we have demonstrated that sPIF (at 50 and 100 nM) increased BCL2 protein level (by 41% and 148%, respectively) and decreased BAX protein level (by 28% and 31%, respectively) in HTR-8/SVneo cells (Figure 6d).

**Figure 7:**
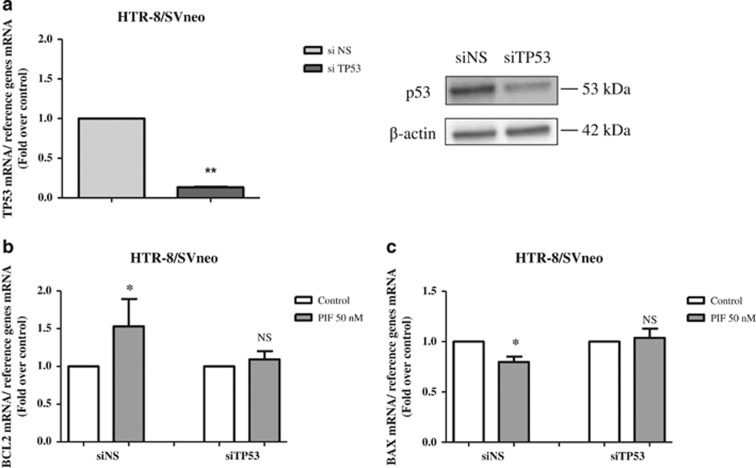
Effect of TP53 silencing in sPIF prevent cell death. HTR-8/SVneo cells were cultured for 48 h in presence of 10 nM siTP53 or 10 nM siNS. Total mRNA were extracted after treatment and quantified by RT-qPCR as described in Materials and methods section. (**a**) TP53 mRNA (left panel) and protein (right panel) levels were measured in transfected cells. Results are the mean±S.E.M. of six separate experiments. (**b** and **c**) HTR-8/SVneo cells transfected with siTP53 or siNS were exposed to sPIF (50 nM). BCL2 (**b**) and BAX (**c**) mRNA expression levels were quantified by RT-qPCR. Results are the mean±S.E.M. of eight separate experiments. *P<0.05; **P<0.01; NS, nonsignificant. Wilcoxon test

To determine whether sPIF anti-apoptotic effect was dependent of p53, we performed invalidation experiments of p53 in HTR-8/SVneo cells using TP53-specific siRNA (siTP53). We then studied the effect of such knocked down on p53 downstream target gene expression. We observed a significant decrease of TP53 mRNA levels (by 86%) confirmed by immunoblot after an invalidation of 48 h (Figure 7a). When TP53 was downregulated, sPIF-induced increase in the expression of BCL2 and reduce in the expression of BAX were negated (Figures 7b and c). The PIF's effects on BAX and BCL2 mRNA expression were conserved in presence of siNS. Furthermore, we have shown that sPIF is able to inhibit apoptosis induced by etoposide (by 49%), an inhibitory of topoisomerase II, which induce p53 dependent apoptosis. Collectively, these result provide evidence that p53 is required for sPIF-mediated modulation of apoptosis.

### PIF protein levels in IUGR and PE placentas

**Figure 8:**
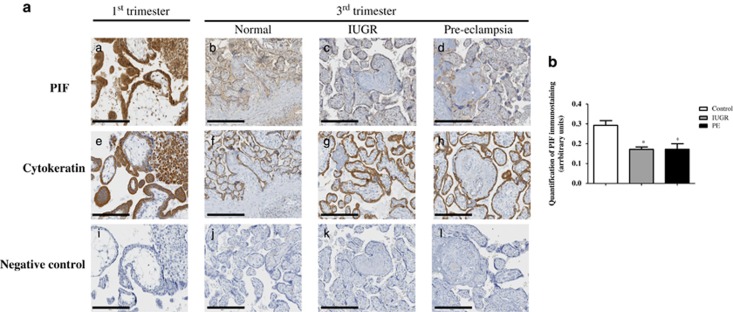
PIF protein levels in human normal, IUGR, and PE placentas. (**a**) PIF protein immunostaining on sections from first-trimester control (*n*=8) (a), third-trimester control (*n*=4) (b), IUGR (*n*=8) (c) and PE (*n*=4) (d) placentas as described in Materials and Methods section. Cytokeratin protein immunostaining was used to identify trophoblastic cells (e-h). The negative control was processed in the absence of primary antibody (i-l). The figure shows one representative among four to eight separate experiments. Magnification, × 20. Scale bar=200 *μ*m. (**b**) Data on the relative quantification of PIF immunostaining are reported as the mean±S.E.M. of four to eight independent experiments. *P<0.05. Mann–Whitney test

To compare PIF expression levels under normal or pathological conditions, we collected human normal, IUGR and PE placentas. Cytokeratin immunostaining was used as a sensible and reliable marker of trophoblastic cells. As shown in Figure 8A, the PIF protein level was lower in normal third-trimester placentas than in first-trimester villi. Moreover, relative quantification of PIF by immunostaining revealed that PIF protein levels were lower in IUGR and PE placentas than in third-trimester control placentas (Figures 8a and b).

## Discussion

The trophoblast serves as a vital interphase between embryo and mother, and so effective invasion and survival in the uterus are required. In this respect, it is noteworthy that (i) the trophoblast has a low antigenic imprint and (ii) the uterus displays an adaptive immune system, in order to prevent rejection of the semi-allogenic fetus. Factors that control this delicate balance have been extensively investigated because they may have a significant therapeutic role in difficult pregnancies.^33, 34^ Among these factors, PIF is a recently identified peptide with activity at the fetomaternal interface. In this context, we performed a genome-wide assessment to better understand the effects of sPIF in the human trophoblast. We tested two different sPIF concentrations (50 and 100 nM), which correspond to the range observed in the maternal circulation^21^ and where sPIF is effective at modulating trophoblast invasion.^20, 21^

Our multifaceted analysis of the signaling involved in sPIF's effect highlighted ‘cell survival', ‘apoptosis' and ‘p53 signaling' pathways, reflecting the presence of robust regulatory control in human trophoblasts.

Our functional experiments effectively demonstrated that sPIF prevents apoptosis in human trophoblasts. More precisely, in EVT, our results revealed that sPIF exerts its anti-apoptotic effect by upregulating BCL2 without any effect on BAX and BAK mRNA expression levels. In HTR-8/SVneo cells, sPIF upregulates BCL2 and downregulates BAX and BAK expression levels. However, the major finding of this study is a decrease of BAX/BLCL2 mRNA ratio in both cells types. As BCL2 and BAX are direct target genes of p53, we performed p53 invalidation experiments in HTR-8/SVneo cells. Our results revealed that silencing the TP53 gene suppressed sPIF's effect on BAX and BCL2 mRNA expression. These data suggest that sPIF prevents cell death, in part, by regulating the intrinsic apoptotic pathway via p53 in human trophoblastic cells. So, we investigated whether p53 expression and transcriptional activity were regulated by sPIF in these cells. In human EVT, we observed that sPIF induces MDM2 mRNA expression without decrease of p53 expression. This atypical result suggest that sPIF could modulate MDM2 transcription by a p53 independent mechanism. A study realized in rat fibroblasts have effectively shown that fibroblast growth factor 1 directly induces MDM2 mRNA expression without p53 implication.^35^ Furthermore, it is well known that phosphorylation of p53 at certain residues (e.g., at Ser-15) is correlated with an increasing transcriptional activity and consequently with the induction of pro-apoptotic gene expression.^36^ We described that sPIF inhibited the p53 transcriptional activity as suggested by the significant reduction of its phosphorylation status. This last result consolidate the anti-apoptotic role of sPIF in human trophoblasts. It will be interesting to determine the effect of sPIF on the major kinase implicated in the p53 phosphorylation (Ser-15), such as ataxia telangiectasia mutated.^37^ Our results agree with those observed in brain and pancreas, where sPIF has an important role in preventing cell death.^16, 38, 39^ In particular, it has recently been reported that the PIF-associated inhibition of the death of mouse neuroblastoma cells is associated with an increase in BCL2 expression and a decrease in microRNA let-7 levels.^38, 39^ Hence, the sPIF's neuroprotective effect may be due (at least in part) to its ability to inhibit the release of a microRNA that is specifically produced after central nervous system damage.^40, 41^ In the field of reproduction, it was recently reported that microRNA let-7 may modulate implantation potential, knockdown of this microRNA in mouse blastocysts completely abolished embryo implantation.^42, 43^ We are currently performing experiments designed to determine microRNA let-7's putative involvement in PIF's actions in human trophoblasts. Consequently, the human placenta provides a novel illustration of sPIF's anti-apoptotic effect. Conversely, it has been described that sPIF stimulates cell apoptosis in human endometrial cells by suppressing BCL2 expression and inducing downstream effects in the BCL2 network.^11^ It is now well established that effective placenta/endometrium dialog is essential for successful embryo implantation. Hence, sPIF may be involved in paracrine communication by simultaneously exerting anti- and pro-apoptotic actions on the placenta and the endometrium, respectively. All these data show that sPIF has a crucial, protective role in the establishment and success of pregnancy.

In initial work, we showed that sPIF promotes the invasive phenotype of human EVT isolated from first-trimester placenta. However, cell invasion is a complex, finely spatiotemporally controlled process that is associated with other mechanisms (such as cell proliferation and immune response).

Our gene array analysis revealed the involvement of a ‘cancer' gene network in the EVT's response to sPIF. More precisely, sPIF treatment was associated with significantly lower mRNA expression of *CECR2* (coding for a transcription factor involved in chromatin remodeling)^44^ and *INHBA* (coding for a ligand in the transforming growth factor-*β* superfamily). Both gene products are involved in cell cycle progression. By controlling trophoblast proliferation and invasion, sPIF may contribute to the control of normal placental development. We also found that sPIF affects the ‘immune response' gene network. Development of tolerance without concomitant immune suppression is essential for effective trophoblast invasion. In this work, the bioinformatics analysis revealed that sPIF induces the mRNA expression of IL17F, a pro-inflammatory cytokine. It was shown that IL17 upregulates trophoblast invasion.^45^ Hence, sPIF may promote trophoblast invasion by also controlling immune response. PIF's immunoregulatory action is well characterized in various human endometrial, neuronal, pancreatic and blood cell types.^11, 15, 46, 47^ In this study, we found that sPIF strongly induces expression of the T cell receptor alpha variable 4 gene (*TRAV4*) in EVT. This finding agrees with a previous study of human endometrial cells, in which sPIF was associated with strong (60-fold) induction of the T-cell receptor alpha.^11^ It seems that sPIF exerts the same immune modulatory effects at the fetomaternal interface by favoring immune tolerance, a key factor for successful pregnancy.

Furthermore, our results showed that sPIF modulates the expression of genes related to the ‘olfactory gene' network. This finding is not surprising, since earlier studies have already demonstrated that olfactory receptor genes are expressed in tissues other than olfactory epithelium (such as heart, testis and more particularly placenta) suggesting that olfactory receptors may have functions other than odorant recognition. Ikatura *et al.*^48^ have even suggested that olfactory receptors may be involved in the trophoblastic invasiveness phenotype. Hence, olfactory receptors may be novel factors involved in sPIF's pro-invasive action on human trophoblasts. We are currently performing additional experiments, in order to better define this hypothesis.

Our genome-wide transcriptome analysis also showed that sPIF upregulates lincRNAs. A recent study demonstrated that the lincRNA MALAT-1 regulates proliferation, apoptosis, migration and invasion in trophoblast cells, and that its underexpression may be involved in the pathogenesis of PE.^49^ Hence, sPIF may regulate placental gene expression (and possibly protein function) through this pathway.

Pregnancies affected by IUGR or PE are associated with elevated perinatal mortality and morbidity rates. PE is a multisystem disorder, characterized by hypertension and proteinuria that affects 2–8% of pregnancies worldwide.^50^ IUGR is characterized by low fetal head circumference, length and weight.^51^ Finally, we found that the level of PIF is relatively lower in the placentas from PE and IUGR as compared with their matched controls. This result suggests that PIF protein levels are disrupted under pathological conditions.

In conclusion, our present results documented a new mechanism for sPIF-mediated trophoblast survival during the first steps in human pregnancy. We also highlighted other sPIF-modulated gene networks related to immune function and tumor suppression that may could complement the PIF's beneficial effects on human placenta. The recognition of sPIF's protective properties by the US Food and Drug Administration has enabled fast-track approval of clinical trials in the treatment of autoimmune hepatitis in non-pregnant subjects. Hence, the use of sPIF could be envisaged in the prevention or treatment of disorders of early pregnancy associated with impaired placentation.^14^

## Materials and methods

### Materials

sPIF analog (purity >95%, as documented by HPLC and mass spectrometry) was produced by Biosynthesis (Lewisville, TX, USA). DMEM-Ham's Nutrient Mix F12 (DMEM/F12), RPMI-1640 medium, penicillin–streptomycin mix, HEPES, MgSO_4_, dimethyl sulfoxide, bovine serum albumin (BSA), Percoll, rabbit monoclonal anti-*β*-actin and DNase type IV were purchased from Sigma Chemical Co. (St. Louis, MO, USA). Matrigel was obtained from BD Biosciences (Le Pont-de-Claix, France) and trypsin was purchased from Difco Laboratories (Detroit, MI, USA). Hank's balanced salt solution and fetal calf serum (FCS) were purchased from Gibco (Invitrogen, Carlsbad, CA, USA). Superscript II RNase H-RT and primers were purchased from Invitrogen and RNAse inhibitor was obtained from AMRESCO (Solon, OH, USA). The Nucleospin RNA kit was obtained from Macherey-Nagel (Düren, Germany). The annexin V-FITC reagent was obtained from Abcam (Cambridge, UK). Precise Tris-glycine gels 4–20% and SuperSignal™ West Pico Chemiluminescent Substrate were obtained from Thermo Fisher Scientific Inc. (Waltham, MA, USA). Mouse monoclonal anti-cytokeratin, pan antibody (KL1) was purchased from Dako (Trappes, France), mouse monoclonal anti-PIF antibody was produced by GenWay (San Diego, CA, USA), rabbit polyclonal anti-phospho-p53 (Ser-15) was purchased from Cell Signaling Technology (Beverly, MA, USA) and mouse monoclonal anti-p53 (DO-1) was purchased from Santa Cruz Biotechnologies Inc. (Santa Cruz, CA, USA). The DAB (3, 3′-diaminobenzidine) detection kit was obtained from Ventana Medical Systems (Tucson, AZ, USA). p53 small-interfering RNA (sip53) were chemically synthesized by Santa Cruz Biotechnologies Inc.

### Tissue collection

First-trimester human placentas were obtained from legal, elective abortions, after 4–12 weeks of gestation (WG), following the provision of written, informed consent. Third-trimester placentas were collected after elective cesarean section. The indications for cesarean section were not related to fetal growth status. The main inclusion criterion for IUGR group was a birth weight below the 10th percentile for gestational age (using growth charts for the French population). PE was defined as the onset of maternal hypertension and proteinuria after 20 week's gestation (systolic blood pressure ≥140 mm Hg; diastolic blood pressure ≥90 mm Hg; proteinuria ≥300 mg per 24 h). This and all other study procedures had been approved by the local investigational review board (Comité Consultatif de Protection des Personnes dans la Recherche Médicale reference: 01–78). Four- to 7-WG placental tissues were obtained after pharmaceutical abortion and 8- to 12-WG placental tissues were obtained after surgical abortion.

### Cell culture

Primary EVT were isolated using an adaptation of the method previously described by Handschuh *et al.*,^52^ and as reported elsewhere.^20^ EVT were seeded onto 5 mg/ml Matrigel-coated dishes containing DMEM/F12 with 1% FCS, penicillin (100 U/ml) and streptomycin (100 *μ*g/ml). The human EVT cell line HTR-8/SVneo, derived from the invasive EVT, was kindly provided by Dr. Nadia Alfaidy (CEA Grenoble, France), in agreement with Dr. Charles Graham (Queen's University, Kingston, ON, Canada). Cells were grown in RPMI-1640 medium with 2% HEPES, penicillin (100 U/ml), streptomycin (100 *μ*g/ml) and 10% FCS.

### Treatment of cultured cells

HTR-8/SVneo cells were cultured in serum-free RPMI-1640 medium for 24 h. EVT were cultured in serum-free DMEM/F12 for 24 h. The next day, media were removed and replaced by serum-free media supplemented with sPIF (50 or 100 nM) or etoposide (42 *μ*M).

### Microarray assays

EVT were cultured in the absence (control) or presence of sPIF (50 nM) for 24 h. Then, total RNA was extracted using the Nucleospin RNA kit's instructions. RNA integrity and purity were checked using a 2100 Bioanalyzer (Agilent Technologies, Massy, France). Four sets of experiments were collected and the expression profile was analyzed with an Agilent® SurePrint G3 Human GE 8 × 60K Microarray (Agilent Technologies, AMADID 039494) using the following dual-color design: sPIF-treated samples were labeled with Cy5 and control samples were labeled with Cy3, using a two-color labeling kit (Low Input Quick Amp Labeling Kit 5190-2306, Agilent Technologies) adapted for small amounts of total RNA (100 ng total RNA per reaction). Hybridization on the microarray was then performed with 825 ng of each linearly amplified and then Cy3- or Cy5-labeled cRNA sample, according to the manufacturer's instructions (Agilent SureHyb Chamber; 1650 ng of labeled extract; 17 h of hybridization; 40 *μ*l per array; 65 °C). After washing in acetonitrile, slides were scanned with an Agilent G2565 C DNA microarray scanner, using the latter's default parameters. Microarray images were analyzed with Feature Extraction software (version 10.7.3.1, Agilent Technologies). Again, the default settings were used.

### Microarray data processing and analysis

Raw data files from Feature Extraction were imported into R using LIMMA^53^ and a R package from the Bioconductor project, and processed as follow: gMedianSignal and rMedianSignal data were imported, control probes were systematically removed, and flagged probes (gIsSaturated, gIsFeatpopnOL, gIsFeatNonUnifOL, rIsSaturated, rIsFeatpopnOL and rIsFeatNonUnifOL) were set to ‘NA'. Intra-array normalization was performed by ‘loess' normalization, followed by quantile normalization of both the Cy3 and Cy5 channels. Next, inter-array difference was normalized by quantile normalization on M values. All probes were then summarized into single genes by taking the mean of all probes that corresponds to the same gene. The missing values were eventually replaced using the KNN algorithm.

Bioinformatics analysis was completed by applying ArrayMining software (http://www.arraymining.net/) and the Cytoscape package. The transcriptome analysis for microarrays was carried out using complementary approaches: a DAVID analysis of the most deregulated genes and a GSEA analysis of enriched pathways (FDR<0.25). The Search Tool for the Retrieval of Interacting Genes (STRING: http://string-db.org/)^54^ was used to screen DEGs for pairs of interacting protein pairs, which were then visualized within the PPI network using Cytoscape (http://cytoscape.org/).^55^ After identifying the DEGs, gene ontology enrichment and KEGG pathway analyses^56, 57^ were performed. The KEGG pathways with *P*-value below 0.05 were selected as enriched functions for the DEGs.

### Quantitative RT-PCR

Total RNA (0.1 *μ*g) was extracted and reverse-transcribed, as previously described.^58^ Quantitative RT-PCR was performed with the primer sets indicated in Table 3 and DyNAmo Flash SYBR green qPCR (Thermo Fisher Scientific Inc.), using a CFX96^TM^ real-time PCR detection instrument (Bio-Rad Laboratories Inc., USA). The second derivative maximum method was used to automatically determine the crossing point (Cp) for individual samples. Three reference genes (ribosomal protein L13A (RPL13A), TATA box binding protein (TBP) and *β*-2 microglobulin (B2M)) were chosen, as previously described.^59^ For each sample, the concentration ratio (target *versus* three reference mRNAs) were calculated using CFX Manager (Version 3.0, Bio-Rad, Munich, Germany) and expressed in arbitrary units. Calibration curves were log-linear over the quantification range, with correlation coefficients (*r*^2^)>0.99 and efficiencies ranging from 1.8 to 2. The PCR products were separated on a 2% agarose gel in 90 mM Tris-borate, 2 mM EDTA buffer pH 8.0, and visualized using GelRed nucleic acid staining and ultraviolet transillumination.

### Cell viability assay

Cell viability was determined in a Trypan blue dye exclusion assay.^60^ Following sPIF treatment, HTR-8/SVneo cells were harvested by trypsinization and incubated for 5 min with 0.04% Trypan blue solution at room temperature. Blue cells (non-viable) *versus* white cells (viable) were counted in a hemocytometer. Cell viability was determined by normalizing the number of white cells against the total number of cells. In parallel, LDH activity^61^ released into the culture media was assayed, as previously described.^62^ The results were expressed per g of total proteins. Protein concentrations were measured according to Bradford's method^63^ using BSA as a standard.

### Annexin V-FITC assay

HTR-8/SVneo cells (1 × 10^6^ cells per well) were plated onto 6-well culture plates. After 48 h of sPIF treatment, cells were stained using an apoptosis detection kit (Abcam), according to the manufacturer's instructions. Flow cytometry analysis of annexin V-FITC staining was performed using an imaging flow cytometer (Amnis/EMD, Imagestream, Merck-Millipore, Darmstadt, Germany). A total of 1000 events were acquired at × 40 magnification, and annexin V-positive cells were quantified using IDEAS 5.0 software (Amnis/EMD, Darmstadt, Germany). Bright field images were used to quantify and verify cell integrity. The apoptosis rate was determined by normalizing annexin V-positive cells against the total number of cells. Etoposide (42 *μ*M) was used as positive control (data not shown).

### TUNEL assay

HTR-8/SVneo cells were cultured at a density of 2x10^5^ cells per well in 12-well culture plates. After 48 h of sPIF treatment, cells were fixed in 4% paraformaldehyde for 20 min at room temperature, washed, and stained using a TUNEL apoptosis kit (*In Situ* Cell Death Detection Kit, Fluorescein, Roche Diagnostics GmbH, Mannheim, Germany) according to the manufacturer's instructions. Cells were counterstained with 1 *μ*g/ml Hoechst33258 (Sigma, St. Louis, MO, USA). Ten fields in each well were imaged using an inverted fluorescence microscope (Olympus, ScanR, Tokyo, Japan), in order to obtain a minimum of 200 cells for analysis. The apoptosis rate was determined by normalizing the number of TUNEL-positive cells against the total number of Hoechst-positive cells, using ScanR software (Olympus, ScanR). Etoposide (42 *μ*M) was used as positive control (data not shown).

### Immunoblotting

HTR-8/SVneo cells (1 × 10^6^ cells per well) were plated onto six-well culture plates. After 48 h of sPIF treatment, cells were lysed in extraction buffer, as previously described.^64^ Twenty micrograms of total protein was resolved by SDS-PAGE (4–20% acrylamide) and transferred onto nitrocellulose membranes using a wet transfer method. After protein transfer, membranes were incubated at 4 °C overnight with rabbit polyclonal anti-phospho-p53 (Ser-15, 1 : 500 dilution, Cell Signaling Technology), mouse monoclonal anti-p53 (DO-1, 1 : 1000 dilution, Santa Cruz Biotechnologies, Inc.), anti-BCL2 (sc-509, 1 : 200 dilution, Santa Cruz Biotechnologies, Inc.), anti-BAX (sc-493, 1:200 dilution, Santa Cruz Biotechnologies, Inc.) or rabbit monoclonal anti *β*-actin (1 : 500 dilution, Sigma). After washing, the membranes were incubated with the peroxidase-coupled secondary antibody for 1 h at room temperature. Blots were developed using the SuperSignal™ West Pico Chemiluminescent Substrate (Thermo Fisher Scientific Inc.).

### TP53 siRNA knockdown

The siRNA for TP53 (p53 siRNA (h): sc29431) was purchased from Santa Cruz Biotechnologies Inc. A fluorescently labeled, non-silencing control siRNA was useful for the optimization of transfection conditions and as a control for nonspecific silencing effects. For the knockdown experiments, HTR-8/SVneo cells were plated in 12-well dishes at 1 × 10^5^ cells per well. Cells were transfected with siRNAs (10 nM per well) using a Lipofectamine RNAiMAX transfection reagent from Invitrogen according to the manufacturer's instructions. sPIF (50) was added 24 h after transfection. After 48 h of culture, the mRNA expression was analyzed by RT-qPCR as described above.

### Immunohistochemistry

Sixteen placental samples (four controls, eight IUGR samples and four PE samples) were processed as previously described.^20^ Digital images of dimethylaminoazobenzene-stained placenta slides were obtained at × 20 magnification using a whole slide scanner (Aperio AT2, Leica Biosystems, Nusslosh, Germany). Specific immunostaining in three representative fields was quantified using the positive pixel count algorithm in Aperio Imagescope software (Leica Biosystems).

### Statistical analysis

Results were analyzed using Graphpad Prism software (version 5.0, GraphPad Software, Inc., La Jolla, CA, USA) and expressed as the mean±S.E.M. of 4–11 separate experiments. Statistical analyses were performed using a non-parametric, paired Wilcoxon's test or a non-parametric Mann–Whitney test. The statistics for assessing the enrichment of group of genes is always based on Chi^2^ contingency tests that are directly performed online by DAVID, STRING or Cytoscape tools. The threshold for statistical significance was set to *P*<0.05.

## Figures and Tables

**Table 1:** List of the top 20 differentially expressed genes after sPIF treatment of EVTs

**Symbol**	**Gene name**	**Fold-change**	***P*****-value**
*Overexpressed genes*			
LOC100507134	LOC100507134 (LOC100507134)	1.652	3.804E–04
AZU1	Azurocidin-1	1.592	6.085E–04
ENST00000390426	T cell receptor alpha variable 4	1.589	1.333E–04
OR10A7	Olfactory receptor, family 10, subfamily A, member 7	1.588	3.348E–04
IL17F	Interleukin 17F	1.554	2.615E–04
XLOC_006051	lincRNA	1.516	4.066E–03
XLOC_004148	lincRNA	1.505	7.676E–04
			
*Underexpressed genes*			
HBG1	Hemoglobin, gamma A	−1.852	5.089E–03
HBE1	Hemoglobin, epsilon 1	−1.633	2.939E–02
RASL10A	RAS-like, family 10, member A	−1.56	2.177E–02
SMOC1	SPARC-related modular calcium binding 1	−1.522	1.819E–05
HBA2	Hemoglobin, alpha 2	−1.487	8.713E–03
PRO2852	Clone FLC0578 PRO2852 mRNA	−1.461	4.171E–03
HBZ	Hemoglobin, zeta	−1.452	3.855E–02
VNN2	Vanin 2	−1.445	4.450E–05
ATG16L2	ATG16 autophagy-related 16-like 2	−1.435	2.155E–02
FRMD4A	FERM domain-containing 4A	−1.432	1.335E–02
GRM4	Glutamate receptor, metabotropic 4	−1.417	1.333E–04
ATP2C2	ATPase, Ca++ transporting, type 2C, member 2	−1.412	2.492E–02
RPLP2	Ribosomal protein, large, P2	−1.408	6.604E–04

**Table 2:** KEGG pathway classification of the top differentially regulated genes after sPIF treatment of EVTs

**KEGG pathway**	**Pathway name**	***P*****-value**
hsa04740	Olfactory transduction	3.61E–04
hsa04080	Neuroactive ligand–receptor interaction	9.32E–05
hsa04060	Cytokine–cytokine receptor interaction	7.50E–04
hsa05200	Pathways in cancer	5.62E–02
hsa05152	Tuberculosis	2.96E–02
hsa05162	Measles	6.13E–03
hsa04020	Calcium signaling pathway	5.41E–02
hsa05142	Chagas disease (American trypanosomiasis)	1.58E–03
hsa04610	Complement and coagulation cascades	6.48E–04
hsa05146	Amoebiasis	1.61E–02
hsa04514	Cell adhesion molecules (CAMs)	5.98E–02
hsa04210	Apoptosis	1.05E–02
hsa05323	Rheumatoid arthritis	1.43E–02
hsa03320	PPAR signaling pathway	8.51E–03
hsa04970	Salivary secretion	1.95E–02
hsa05332	Graft-*versus*-host disease	5.25E–04
hsa04940	Type I diabetes mellitus	8.97E–04
hsa04672	Intestinal immune network for IgA production	2.59E–03
hsa05144	Malaria	2.59E–03
hsa00590	Arachidonic acid metabolism	9.36E–03
hsa00980	Metabolism of xenobiotics by cytochrome P450	2.10E–02
hsa04971	Gastric acid secretion	2.65E–02
hsa05320	Autoimmune thyroid disease	1.24E–02
hsa00830	Retinol metabolism	2.87E–02
hsa04115	p53 signaling pathway	4.86E–02

**Table 3:** Primers used for PCR

**Primer set**	**Sequence**	**PCR product (pb)**
*AZU1*		
Sense	5′-CCCCTTTTGGACATCGTTGG-3′	159
Antisense	3′-CCCGGGGTTCTGGCTTTG-5′	
*HBG1*		
Sense	5′-CACTCGCTTCTGGAACGTCT-3′	161
Antisense	3′-GGTAGACAACCAGGAGCCTTC-5′	
*HBE1*		
Sense	5′-CTTCAAGCTCCTGGGTAACG-3′	174
Antisense	3′-GTCAGGGTCACAGGAACACC-5′	
*TP53*		
Sense	5′-ACTAAGCGAGCACTGCCCAA-3′	231
Antisense	3′-CAGTCAGATGGAGGGCGGTA-5′	
*BCL-2*		
Sense	5′-ATGTGTGTGGAGAGCGTCAACC-3′	196
Antisense	3′-CCGACAGAGTCATCTGAGGCATGA-5′	
*BAX*		
Sense	5′-CAAACTGGTGCTCAAGGCC-3′	188
Antisense	3′-TAGAAACACCGCCCTCACG-5′	
*BAK*		
Sense	5′-CCATTCCTGGAAACTGGGCT-3′	125
Antisense	3′-CCGTCCGACTAGGGCAG-5′	
*MDM2*		
Sense	5′-GTGAAGGAAACTGGGGAGTCTT-3′	101
Antisense	3′-ACGTTATGGTTTTACAGACATGGA-5′	
*TBP*		
Sense	5′-TGCACAGGAGCCAAGAGTGAA-3′	132
Antisense	3′-CACATCACAGCTCCCCACCA-5′	
*B2-MICROGLOBULIN*		
Sense	5′-TGCTGTCTCCATGTTTGATGTATCT-3′	86
Antisense	3′-TCTCTGCTCCCCACCTCTAAGT-5′	
*RPL13A*		
Sense	5′-CCTGGAGGAGAAGAGGAAAGAGA-3′	125
Antisense	3′-TTGAGGACCTCTGTGTATTTGTCAA-5′	
